# Potential of DNMT and its Epigenetic Regulation for Lung Cancer Therapy

**DOI:** 10.2174/138920209788920994

**Published:** 2009-08

**Authors:** Mingqing Tang, William Xu, Qizhao Wang, Weidong Xiao, Ruian Xu

**Affiliations:** 1 Engineering Research Center of Molecular Medicine, Ministry of Education, 362021, China & Institute of Molecular Medicine, Huaqiao University, Fujian, 362021, China; 2 Faculty of Science, University of New South Wales, 2052, Australia; 3 Department of Pediatrics, University of Pennsylvania, Philadelphia, PA 19104, USA

**Keywords:** Lung cancer, epigenetic regulation, DNA methylation, DNMT, gene silence, epigenetic therapy.

## Abstract

Lung cancer, the leading cause of mortality in both men and women in the United States, is largely diagnosed at its advanced stages that there are no effective therapeutic alternatives. Although tobacco smoking is the well established cause of lung cancer, the underlying mechanism for lung tumorigenesis remains poorly understood. An important event in tumor development appears to be the epigenetic alterations, especially the change of DNA methylation patterns, which induce the most tumor suppressor gene silence. In one scenario, DNA methyltransferase (DNMT) that is responsible for DNA methylation accounts for the major epigenetic maintenance and alternation. In another scenario, DNMT itself is regulated by the environment carcinogens (smoke), epigenetic and genetic information. DNMT not only plays a pivotal role in lung tumorigenesis, but also is a promising molecular bio-marker for early lung cancer diagnosis and therapy. Therefore the elucidation of the DNMT and its related epigenetic regulation in lung cancer is of great importance, which may expedite the overcome of lung cancer.

## 1. INTRODUCTION

Lung cancer is the leading cause of mortality in both men and women worldwide. Currently, one in four deaths in America is owed to cancer. In 2008, there were 215,020 new cases of lung cancer, accounting for 15.0% of all new cancer cases; estimated death rate of lung cancer is expected to account for 28.6% of all cancer deaths [1,2].

**Fig. (1):**
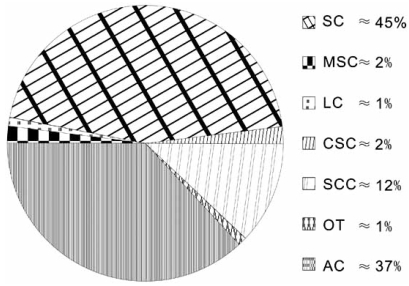
**Types and percentages of lung cancer.** SC: squamous lung cancer; AC: adenocarcinomas; LC: large cell undifferentiated carcinomas; SCC: small cell carcinomas; MSC: mixed small cell carcinomas; CSC: combined small cell carcinomas; OT: other types.

Lung cancer can be divided into two major histopathological groups: non-small-cell lung cancer (NSCLC) and small-cell lung cancer (SCLC) (Fig. **1**). About 80%~85% of lung cancers are NSCLC, which can be subdivided into adenocarcinomas, squamous cell, and large-cell undifferentiated carcinoma. Squamous cell carcinoma (SC) and adenocarcinoma (AC) are the most prominent types. SCLC accounts for just 15%~20% of all lung cancers and is far more aggressive than NSCLC. SCLC is split into different types depending on which cells in the lungs are affected by the cancer: small cell carcinoma (the most prominent type), mixed small cell carcinoma and combined small cell carcinoma. Besides the two major types of lung cancer there are other types of tumor which can appear in the lungs, and some of these are benign [2].

Epigenetics refers to a change in gene that is heritable (i.e. can be passed on through cell division) but is not involved in a change in DNA sequence. This is in contrast with true genetic alterations [3-5]. Epigenetic processes include genomic imprinting [6], gene silencing [7,8], x-chromosome inactivation [9], reprogramming in transferred nuclei [10, 11] and some elements of carcinogenesis [12]. Epigenetic mechanisms play a crucial role in regulation of gene expression by affecting chromatin accessibility. DNA methylation catalyzed by specific DNA methyltransferase (DNMT) represents an important mechanism for the epigenetic control of gene expression and the maintenance of genome integrity [13].

This review focuses on the up and down stream of DNA methylations, which occurs at the 5-position of cytosine in a CpG dinucleotide context. Normally, 99% sequence of the entire genome is rarely with CpG site, only about 1% sequence is CpG rich, so-called CpG islands. Human genome contains 29000 CpG islands [14] and approximately half the CpG islands were often associated with promoter regions, and half the promoter regions in human genome possess these islands [15]. As for the non-CpG islands containing promoters, they bear other methylation patterns. Accumulating investigations have demonstrated that lung cancer development is linked with aberrant DNA methylation patterns, which can be characterized as global genome hypomethylation accompanied by regional hypermethylation [16-18]. While global genome hypomethylation may be associated with the induction of chromosome instability (a cellular state characterized by an increased rate of genetic changes, including DNA sequence changes, aneuploidy, chromosome translocations, and/or gene amplication), gene-specific hypermethylation is known to be associated with the inactivation of various pathways involved in tumorigenic process inducing DNA repair, cell cycle regulation, inflammatory/stress response and apoptosis, thus the aberrant methylation patterns play a key role in epigenetic regulation of lung tumorigenesis [19-22]. At the moment, more and more researchers focus on the DNMT regulation area, and the proposal and carrying out of Epigenomic Project (contrast to the Human Genome Project) has put the epigenetic mechanism of lung tumorigenesis to a such hot status, and surely expedite the clarification of DNMTs induced epigenetic disturbance mechanism for lung tumorigenesis and progression.

## 2. OVERVIEW OF DNMT

### 2.1. Classification

To date, multiple DNMTs appear to be present in human, with varying degrees of specificity toward unmethylated and methylated DNA substrates, including DNMT1 (gene aliases: *CXXC9*,* DNMT*, *FLJ16293*, *MCMT*, *MGC104992*) [23], DNMT2 (gene aliases: *RP11-406H21.1*, *TRDMT1*, *M.HsaIIP*, *PuMet, RNMT1*) [24], DNMT3A (gene aliases: *DNMT3A2*, *M.HsaIIIA*), DNMT3B (gene aliases: *ICF, M.HsaIIIB*) [25] and DNMT3L [26].

### 2.2. Structure

**Fig. (2):**
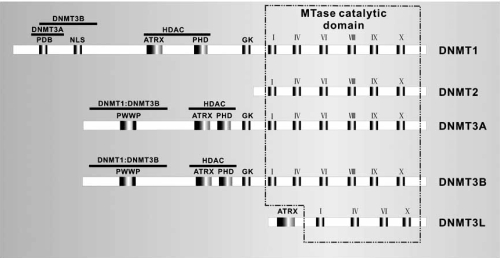
**Structural and the functional domain of DNMTs.** PDB: proliferating cell nuclear antigen (PCNA) binding domain; NLS: nuclear localization signal sequence, is responsible for localization of DNMTs in the nucleus; ATRX: cysteine alpha thalassemia retardation on the X rich zinc finger DNA binding motif; PHD: polybromo homology domain, targeting DNMTs to the replication foci; GK: GK-rich repeats; MTase: methyltransferase; PWWP tetrapeptide is only present in N-terminal domains of DNMT3A and DNMT3B; The C-terminal domain contains six conservative motifs I, IV, VI, VIII, IX and X. Mapped interactions with DNMT3A, DNMT3B, PCNA, histone deacetylase (HDAC)1 and -2 are shown above the diagram.

Structurally, all DNMTs share a common catalytic domain in the carboxyl terminus, which consists of several α-helical and β-sheet structures. This catalytic domain is characterized by the presence of six conserved amino acid motifs, namely I, IV, VI, VIII, IX and X. Motif I and X are filed together to form the most of the binding site for methyl donor (S-adenosyl-L-methionine, SAM). Motif IV contains the prolylcysteinyl dipeptide that provides the thiolate at the active site. Motif VI contains the glutamyl residue that protonates the 3 position of target cytosine. Motif IX has a role in maintaining the structure of the target recognition domain, usually located between motif VIII and IX [27,18]. The N-terminal domains of DNMT3A and -3B exhibit some homology, but they differ significantly with N-terminal domains of DNMT1 [28]. The N-terminal regulatory and the C-terminal catalytic domains were linked by a short fragment of repeated GK dipeptides. DNMT3L was assigned to the DNMT3 family on the basis of the high conservation of its N-terminal PHD-like zinc finger domain with the corresponding domains of DNMT3A and -3B. The C-terminus of DNMT3L also show partial homology to the C-terminal catalytic regions of DNMT3A and -3B, although key catalytic residues involved in the transfer of the methyl groups are not conserved in DNMT3L [29,30]. The unique terminal domains of mammalian DNMTs harbor several regulatory domains that mediate both protein-protein and protein-DNA interaction (Fig. **2**) [27].

### 2.3. Biological Functions

#### 2.3.1. DNMT1 Family

Functionally, DNMT1, the 1st identified DNMT, is the primary enzyme responsible for copping methylation patterns after DNA replication because it localizes to replication loci and it has a 7- to 21- fold preference for hemimethylated DNA substrates than unmethylated DNA substrates, thus this protein is referred to as a maintenance methyltransferase (i.e., copies the methylation patterns of the parental strand to the daughter strand during DNA duplication) [31,32]. Interestingly, recent studies have associated DNMT1 with methylation of unmethylated human CpG islands in cancer cells. They reported that a majority of the *de novo* methyltransferase activity was provided by DNMT1 with gene-specific preference, charging the previous knowledge of DNMT1. Then they substantiated the specificity of DNMT1 was not inherent to the enzyme but may be due to associated cellular factors [33]. And the finding that DNMT1-mediated suppression of the unmethylated rDNA promoter involves *de novo* methylation of the promoter could further substantiate the *de novo* methylation activities of DNMT1 [34]. Many researchers hold that DNMT1 activity is required for *de novo* methylation at non- CpG cytosines, and perhaps to an extent even in CpG islands [35,33]. In addition to methyltransferase activity, interaction with DNMT1-associated protein (DMAP), E2F1, HDAC and methyl-CpG binding proteins (MBD) make DNMT1 a crucial element of transcription suppression complex [36,37].

#### 2.3.2. DNMT2 Family

A summary to the previous observations on DNMT2 family, DNMT2 does not methylate DNA but instead methylates small RNA. Mass spectrometry showed that this RNA is aspartic acid transfer RNA (tRNA (Asp); TRD) and that DNMT2 specifically methylates cytosine-38 in the anticodon loop, and the function of DNMT2 was highly conserved [38,39]. Importantly, Hermann *et al*. provided compelling evidence that DNMT2 has some de novo CpG methylating capacity [40].

#### 2.3.3. DNMT3 Family

DNMT3A and -3B are essential for early embryonic development and the establishment of repressive complex. Because DNMT3A and -3B have similar affinities for both unmethylated and hemi-methylated DNA substrates, their function as the only bona fide* de novo* DNMT (i.e. to form specific methylation patterns in the unmethylated strand without any models) to affect the methylation status of normally unmethylated CpG sites and to recruit HDAC to chromatin [41]. However, there were studies showing both DNMT1 and DNMT3 exhibit some levels of both maintenance and *de novo *methylation activity *in vitro*, suggesting that this classification of the DNMT may be over simplified [26]. Recently, studies by Geiman and coworkers found a role for DNMT3B in maintaining genomic stability independent of its enzymatic activity. This could potentially be explained by the participation of DNMT3B in the condensation complex, which is involved in proper segregation of sister chromatids during mitosis [42]. DNMT3A2, a shorter isoform of DNMT3A, is also required for genomic imprinting [43]. DNMT3A2 and DNMT3B, along with the four core histones, were identified as the main *in vivo* interaction partners of epitone-tagged DNMT3L [44].

**Fig. (3). Models of DNMT associated methylation and gene silence.**
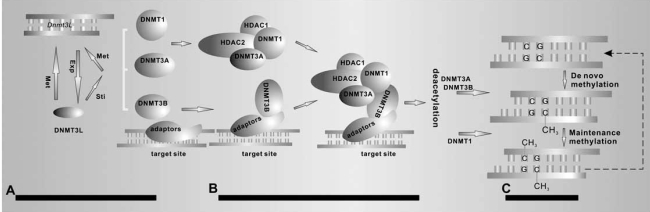
**(A)** Auto-regulation of DNMTs. DNMT1, DNMT3s all could methylate (Met) the Dnmt3L gene, thus the DNMT3L expression (Exp) is repressed. On the other hand, the DNMT3L could stimulate (Sti) the methyltransferase activity of DNMT1, DNMT3A and -3B. **(B)** Mechanism that DNMT caused gene methylation. First, certain adaptors (HP1, lymphoid-specific helicase (LSH)) recognize the specific gene site, and serve as a scaffold protein to recruit DNMT3B. Second, the DNMT1 makes an interaction with DNMT3A and HDACs, they then form the repression complex via the DNMT3B, and HDACs remove the acetyl from the histone tails. Finally, the DNMTs behave their methyltransferase activity, making the gene methylated. **(C)** DNMT3A and -3B are the de novo methyltransferase, the DNMT1 maintains the methylation status, while the methylated genes are often silenced. This methylation process could be reversed by some demethylation substrates.

DNMT3L, a DNMT3A and -3B like protein, is inactive on its own, but DNMT3L plays a key role in allowing DNA methylation during the maturation of germ cells. In theory, DNMT3L could ‘regulate’ other active DNA methyltransferases or could target DNA methylation to certain areas, such as imprinting centers [45,46]. Some data suggest that DNMT3L may be a probe of histone H3 lysine 4 (H3K4) methylation, and if the methylation is absent, then DNMT3L could induce *de novo* DNA methyalion by docking activated DNMT3A2 to the nucleosome, which indicates that DNMT3L might function together with these two *de novo* DNA methyltransferases [44,47]. DNMT3L is the first stimulatory factor for DNA methylation to be described. *Dnmt3L* is controlled *via *its promoter methylation during embryonic development. Genetic studies showed that DNMT3A, -3B and -3L are all involved in the methylation of the *Dnmt3L* promoter. Interestingly, DNMT3L also contributes to the methylation of its own promoter in embryonic development. We therefore can propose an auto-regulatory mechanism for the control of DNA methylation activity whereby the activity of the *Dnmt3L* promoter is epigenetically modulated by the methylation machinery including DNMT3L itself (Fig. **3A**) [29].

*In vitro* methylation assays have shown that DNMT3 family could cooperate with DNMT1 to extend methylation, and DNMT1, DNMT3 could bind HDAC and medicate formation of repression complex surrounding the certain promoter region, because of the HDAC binding motif in their structures (Fig. **2**). Mostly, it’s acknowledged that the normal methylation patterns was established by DNMT1 cooperated with DNMT3 family, the maintenance function of DNMT1 methylation guarantees the initiation of DNMT3 *de novo* methylation, the DNMT3 elevates the methylation level to the wanted level [27,32]. In a word, DNMTs play an essential role in epigenetics, which control the DNA methylation status at level.

Since the diverse roles, functions, activities of DNMTs have being reported, it’s therefore reasonable to speculate that the *de novo *or maintenance function is cell lines, gene sequence and cellular setting specifically, thus when the experimental conditions come to different material, different target, the diverse results may be emerging.

## 3. DISREGULATED DNMT CORRELATED WITH LUNG TUMORIGENESIS

### 3.1. Lung Tumorigenesis Induced by Disrupting Cell Cycle and its Inner Balance

DNMT1 and DNMT3B, localized in the nucleolus, could synergistically maintain the methylation profile of the human rDNA promoter and regulate its expression. However DNMT3B represses the rDNA promoter activity by a methylation independent mechanism, which is different from that of DNMT1. Thus the DNMTs could regulate the rRNA level, ribosomes synthesis and cell cycle. This is consistent with the finding that reduced methylation of the rDNA promoter in some primary carcinomas relative to matching control tissues [34]. On the other hand, the over-expression of DNMTs can lead to ribosomal DNA (rDNA) hypermethylation, subsequently affect the methylation of ribosomal RNA (rRNA) at 2’-O position. A pre-rRNA must undergo maturation to form a functional rRNA, and the pre-rRNA would be degraded during this process if the 2’-O position is non-methylated. If so, the functional ribosome can’t be biosynthesized, and the cell-cycle would become arrested once without the functional ribosome, therefore the DNMT statistically affects cell proliferation, i.e. the higher DNMT activity, the higher speed of cell proliferation [48]. In another study, when the level of DNMT1, DNMT3A and -3B in lung cancer cell lines were normalized against PCNA, no over-expression of DNMTs were observed, suggesting overexpression of some of these genes may be a reflection of increased cell proliferation [49]. To our knowledge, DNMT1, not only affects cell cycle, but is also regulated by cell cycle *via *the pRB/E2F pathway. More recently, a study investigated cell cycle-specific gene expression indentified DNMT1 as part of a G1-S cycle cluster [50]. Kishikawa *et al*. through investigating the expression of *Dnmt1*, and found that the control elements (e.g. SP1, SP3, P300) of *Dnmt1* were mainly recruited at G1, S phase respectively, which coordinately regulated the expression of *Dnmt1* at S phase. These data suggested that *Dnmt1* was regulated in cell-cycle dependent manner [51]. Thus, there are considerable evidences to support the cell cycle-specific regulation of DNMT1 [52]. Although some results conflicted with the view that cell proliferation was inversely associated with differentiation [53], most studies available so far were consist with this conclusion. Hence, DNMT over-expression not only blocks normal differentiation progress but also assists proliferation. Since there is a homeostatic balance, i.e. proliferation and differentiation in each cell, once this balance is disrupted, the cell growth becomes out of control, which lead to tumor formation. During tumorigenesis, when cells express a specific protein, which can interact with DNMT and stabilize it. From then on, the cell is able to secrete self-stimulus and becomes exo-stimulus independent, cell differentiation is further repressed.

### 3.2. Lung Tumorigenesis Induced by Silencing TSGs

Although it is well established that the predominant consequence of methylation is gene silence, it is less clear if this is mediated directly or indirectly [54,55]. The direct inhibition involves interactions of methylated DNA with methylation-sensitive factors (E2F, CREB, AP2, cMyc/Myn, NF-kB, cMyb, ETs), disabling their DNA-binding ability, and repression of transcription [54,55]. In addition, methylated DNA recruits m^5^CpG-binding proteins (MeCP) and m^5^CpG-binding domain proteins (MBD). MeCP1 and -2 bind specifically to methylated DNA in whole genome, and form spatial obstacles that are unable to bind transcription factors (TFs) to promoter sequences. MBD protein family, including MBD1, MBD2, MBD3, MBD4, and uncharacterized Kaiso complex, binds to methylated DNA [56,57]. While an indirect repression may be involved in MBD containing proteins, the MBD-CpG complex recruiting HDAC, resulting in a deacetlyated repressive chromatin structure [58,59]. Recent findings suggest that a full picture was much more complex, with cancer-specific DNA hypermethylation (associated with histone modification) affecting whole gene ‘neighborhoods’ up to an entire chromosome band [60]. More recently, as a result of aberrant histone methylation without DNA methylation, a novel mechanism of cancer-specific loss of expression of neighboring genes has been reported [61].

It is only now that the nature of DNA hypermethylation induced gene silencing start to be understood, which is mediated by a series of events that include methylation of cytosines within the gene promoter and the establishment of heterochromatin in which the histone tails are modified through effects on acelylation, phosphorylation, methylation and ubiquitylation [62]. There is an agreement on the universal coexistence of both histone modification and DNA methylation at silenced gene and Cross-talk between these epigenetic mechanisms during gene silencing [63,64]. However, there is no concensus on which epigenetic mechanism initiates and steers this communication. In one scenario, DNA methylation may be the primary marker for gene silencing that triggers events leading to non-permissive chromatin state [65]. The process involved through the MePC2, HDAC were recruited to the methylated DNA, resulting in a deacetylated repressive chromatin structure [58]. In another scenario, CpG methylation was not a primary cause of initiation of transcription inactivation, but maintained long-term silencing of genes that have already been switch off by other mechanisms [66-69]. From the subtle relationship between DNA methylation and histone modification, some studies indicated that firstly, the epigenetic information could flow from histone to DNA through histone deacetylation and DNA methylation [70]. DNA methylation might exert a positive feedback to histone modification, so the epigenetic information could come back to histone [71]. To explain the mechanism of DNA methylation coordinated histone modification in gene silencing, it has been proposed a self-reinforcing epigenetic cycle model that maintains and perpetuates a repressed chromatin state: Firstly, methylated histone tails interact with adaptor protein HP1, LSH, leading to DNMT3B recruiting. Secondly, through the directly binding of LSH with DNMT3B, HDAC and DNMT1 are recruited to the site, and forming the large protein complex. Thirdly, the recruited HDAC deacetylate histone tails, initiating the transitional transcriptional repression. Then, the more DNMTs recruited, the higher concentration of DNMTs, and cause the specific methylation to keep the gene silence. Eventually, MBD-containing proteins bind to methyl-CpG site (mCpG), and mCpG-MBD complexes interact with HP1, recruiting histone-lysine methyltransferase (HKMT), re-methylating the histone [72]. This model has indicated that DNA methylation, histone deacetylation and histone methylation work in a cooperative manner to enhance and maintain the epigenetic regulation, keeping the target gene in a semi-permanent silence state (Fig. **3B**, **C**). Other than the methylation mediated gene silence, the relatively large N-terminal domains of DNMTs can also mediate transcriptional repression of genes independent of their methyltransferase activity [73,36,37].

In lung cancer, several sets of genes including the tumor suppressor gene (TSG) have been shown to be frequently methylated and inactivated (Table **1**).

### 3.3. Lung Tumorigenesis Induced by Causing Epigenetic and Genetic Changes

In fact, DNA methylation is a more stable epigenetic modification compared with other epigenetic patterns. In successive generations of an Arabidopsis mutant lacking the maintenance of methylation at CpG dinucleotides (mCpG), Mathieu *et al*. found that the loss of mCpG triggers genome-wide activation of alternative epigenetic mechanisms. These compensatory responses act in a stochastic fashion and lead to the accumulation of aberrant epigenetic patterns. Latterly, Karpf and colleagues investigated the potential link between DNA hypomethylation and/or DNMT loss and genomic instability in human cancer cells, and presented a number of novel findings, one of which showed that DNMT loss resulted in bona fide chromosomal instability [97]. These results suggest that mCpG might provide not only direct epigenetic regulation but also coordinate and stabilize epigenetic memory required for faithful replication [98]. Transcriptional silencing by CpG island hypermethylation rivals genetic changes as a critical trigger for neoplastic development and progression [99]. DNMT induced epigenetic alterations may arise in any stage of tumor development, but recent studies have established that these alterations occur mostly in the precancerous stage. Early epigenetic changes might then lead to the genetic alterations, and all these changes could drive cancer formation [100]. For example, the aberrant DNA methylation, silencing the O-6-methylguanine-DNA-methyltransfease (MGMT) gene, left the cell unable to directly remove adducts from the O position of guanine, and further contributes to genetic alterations. Furthermore, DNMT can assist methylated cytosine deamination (i.e., cause the C to G transition mutation). When DNA duplicated, this mutation induced a G: C to A: T mutation, which could cause genetic alterations [101]. Consequently, both epigenetic alterations and genetic changes are important throughout cancer development. At the initial stages of tumorigenesis, epigenetic alterations enable the cells to form tumor-like clones and induce genetic alterations. Subsequent tumor development and metastasis progress are not only dependent on genetic mutation but also the accumulation of epigenetic alterations. In lung cancer, one of the most consistent genetic abnormalities is the loss of the short arm of chromosome 3, the hyperproliferation of chromosome 1 and 12. The loss of 3p alleles was observed in >90% of SCLC and approximately 50% of NSCLC. Some tumor suppressor genes such as *RASSF1A*, on 3p21, are absent in all SCLC and in 65% of NSCLC. Other candidates at the 3p are *FHIT* [102], *beta-catenin* [103], *RARB* [83], *CAV1* (caveolin-1) [104]. Hence, tumorigenesis is the outcome of epigenetic alterations in cooperation with genetic alterations.

### 3.4. Which DNMT is Dominant for Inducing Lung Tumorigenesis

The over-expression of DNMT1 is an early indicator in the development of lung cancer, which occurs earlier than the methylation disturbance. This conclusion was first implied in studies where only a 2-fold over-expression of the *DNMT1* gene in NIH3T cells resulted in a marked increase in overall DNA methylation and tumorigenic transformation [105]. There are also reports that the high DNMT1 activity would promote tumor cell proliferation. The RNA interference-based knockdown experiment in NSCLC cell line A549 has provided evidence that DNMT1 level correlates with the A549 proliferation ability and clone forming ability. The exact mechanism is still poorly known, which is maybe related to P21 expression [106]. The hypermethylation of TSG is a common thing in lung cancer, and it is generally acknowledged that DNMT1 is correlated with hypermethylation in the TSG promoters, especially among smoking SCC patients. Suzuki *et al*. employed siRNA to down-regulate the DNMT1 expression in NSCLC cell line NCIH1299, and resulted in >80% reduction of promoter methylation in* RASSF1A, CDKN2A (alias P16/INK4a), CDH1 *and* HPP1* gene. They also observed that the reactivation of methylation-silenced gene after treatment [78]. However, some experiments showed that simply down regulated DNMT1 expression was unable to reverse the DNA methylation and reactivate the TSG [107]. In addition, a poor prognostic trend was found for patients with highly expressed DNMT1 protein and the association was apparent in SCC patients [65]. Similar results were obtained by using Cox proportional hazards regression analysis to determine whether elevated mRNA levels of *Dnmt* were an independent prognostic factor, and it is found that deregulation of DNMT1 was an independent prognostic factor in NSCLC, and the elevated DNMT3B did not affect patient prognosis [108].

It is also commonly acknowledged that DNMT3B correlated with TSG hypermethylation in lung cancer. But many conflicting results have emerged [107]. The elevated mRNA levels of *Dnmt3b* were not significantly associated with hypermethylation of the six TSGs (*p16, RARβ2, H-Cadherin, GSTP1, RIZ *and* FHIT*), thereby suggesting that other factors might be involved in CpG island hypermethylation of TSG in a gene-specific basis in primary NSCLC [108]. While another study showed that *Dnmt3b* knockdown could arrest lung cancer cell growth, assist apoptosis and re-activate the TSG, which has been silenced due to hypermethylation [101]. This study also claimed DNMT3B was essential for lung tumorigenesis and progression.

DNMTs should interact with other active factors to perform their biological functions *in vivo*. An interaction between members of DNMTs family has been well established, in which is normally in a cooperative manner, DNMT1 is dominant, while DNMT3B assists and cooperates with DNMT1 to pay its role in lung tumorigenesis [65].

### 3.5. The Chief Culprit, Smoke, Induces Lung Tumorigenesis Mainly Through DNMT

In addition to chronic inflammation and/or persistent infection with pathogenic microorganisms, cigarette smoking is another major factor associated with alternations of DNA methylation during multistage lung tumorigenesis [109,18]. Several animal models and human NSCLC samples show that cigarette smoke leads to high level DNMT activity [110,111]. In contrast, AC is often, especially in women, not associated with cigarette smoke, and promoter hypermethylation of the *MGMT* gene is more common in AC in non-smokers than smokers, furthermore, epidemiologic data also suggest that male and female patients might have different susceptibilities to tobacco carcinogens [112,113]. To elucidate the phenomenon that DNMT was highly over expressed in smokers with lung cancer, some reports have shown that tobacco components stimulated Ap1, Akt and NFkB–dependent signaling pathway in lung cells, and the Ras-Ap1 signaling pathway could enhance the DNMT expression [114-116]. Exposure to tobacco smoke may induce selective changes in a limited set of key regulatory transcription factors, including SP1 protein, the Cis-acting factor that normally protects the islands from methylation [117]. The over expression of DNMT leads to the promoter and 5’ flanking regulation region methylation of MGMT gene, which down-regulates the expression of MGMT protein, a direct DNA repair enzyme that protects cells from the carcinogenic effect of alkylating agents in cigarettes, by removing adducts from the O6 position of guanine [118]. If MGMT is hypoexpressive or inactivated, DNA lesion could not be corrected, the G:C to A:T mutation will form, which contributes to the formation of lung cancer. Many experiments have confirmed that lung tumorigenesis was significantly correlated with the activity of MGMT [119]. In light of the mechanism that smokes cause lung cancer, Lemjabbar and colleagues have demonstrated that tobacco smoking could activate the Wnt, Hh pathway, which play an essential role in lung tumorigenesis [120]. Other studies have shown that tobacco smoke causes genetic alterations such as the loss of chromosome 3, where is loci of abundant TSGs [121].

## 4. NOVEL LUNG CANCER DIGNOSIS STRATEGY BY METHYLATION PROFILES

It was well demonstrated that DNA methylation patterns play a key role in lung tumorigenesis [16,110]. Distinct methylation patterns have provided molecular distinctions between different histological subtypes of lung cancer. (i.e., cancers from different organs display distinct methylation profiles), even different histological subtypes of cancers within a given organ have appeared to have distinct methylation profiles. DNA methylation profiles of both normal and cancer tissues tend to be organ-specific, and hot spots of DNA hypermethylation may reflect the diversity of carcinogenetic factors. This has been illustrated by the recent analysis of DNA methylation levels in 91 lung cancer cell lines: 7 out of the 23 CpG island loci tested showed a significant difference in the methylation values between SCLC and NSCLC cell lines [122]. This was further supported by the results of Tsou and colleagues, who examined the DNA methylation status of the 14 loci in 6 malignant mesothelioma (MM) tissue samples, 7 AC tissue samples, 11 non-tumor lung tissue samples, 8 AC cell lines and 10 MM cell lines and gave a similar outcome [123]. When using DNA methylation profiles as indicators to estimate carcinogenetic risk, elimination of such etiological factors may be efficient for cancer prevention. Consequently, early diagnosis of cancers using DNA methylation profiles as indicators may be a promising avenue. Recently developed technologies for accessing genome wide DNA methylation status will be useful to identify the DNA methylation profile, which is the optimum indicator of prognosis. The distinct profile therefore may serve as a novel strategy for lung cancer diagnoses and therapy.

Multiple genes often intensively methylated in SCLC are* RASSF1A* [78], *hsRBC* [83], *CAV1* [104] and *RARB* [124]. In contrast, the frequencies of methylation of *MGMT* [112], *PAX5-alpha, PAX5-beta* [125], *TSLC1* [126], *hMLH1, hMLH2* [127],* DAPK* [128], *P14IRF* [129], *FHIT* [130],* beta-Catenin* [80] are remarkably higher in NSCLC than SCLC. The *P16* gene is more frequently methylated in SCC than AC, but the *APC* [123], *CDH13* [131] are seemly reversed. Others liable to methylation in lung cancer are shown in Table **1**.

## 5. DNMT RELATED EPIGENETIC CANCER THERAPY

### 5.1. Overview

Despite of new drugs and therapeutic regiments, the prognosis for lung cancer patients has not significantly changed in the last 20 years. Surgery remains the main therapy for patients, but large fraction of patients cannot undergo curative resection. Innovative therapeutic strategies are urgently needed for lung cancer treatment. Because of the reversibility of epigenetic events, the pharmacological agents that can reverse this epigenetically mediated progress make it an ideal target for prevention and therapy [132,133]. Epigentically active drugs currently within clinical trials include histone deacetylase inhibitors (HDACi) and DNMT inhibitors (DNMTi) [134], and the most extensively studied are DNMTi [135]. Considerable promise lies in the further development of DNMT targeting therapies that already have shown antitumorigenic effects for lung cancer and several malignancies [136,137] Table **2**.

### 5.2. Nucleoside DNMTi

Among the sea of DNA demethylating agents, the most widely used in experimental and clinical scenarios is the nucleoside DNMTi, which mainly comprise cytosine analogs and cytidine deaminase analogs, including 5-Aza-cytidine (5-Aza-CR, azcitidine, Vidaza), 5-Aza-2’-deoxycytidine (5-Aza-CdR, decitabine, Dacogen), 1-β-D-arabinofuranosil-5-azacytosine (fazarabine) and 1-β-D-ribofuranosyl-2 (1H)-pyrimidinone (zebularine) [4, 138-140]. The archetypal DNMTi 5-Aza-CR is a simple derivative of the nucleoside cytidine, and its demethylating activity has been reported 30 years ago [141]. Today, 5-Aza-CR has been approved by the Food and Drug Administration (FDA) as an antitumor agent for the treatment of myelodysplastic syndrome (MDS) [135]. And in prostate cancer cell lines PC3, Dul45 and LNCap, growth arrest and DNA damage inducible, alpha (GADD45α) was upregulated by the treatment of DNMTi (5-Aza-CR) and confer sensitivity to chemotherapy, which represent a potential way for treatment of prostate cancer [142]. Schmitz further reveal that the GADD45αprotein also bare demethylating ability, when it target to the DNA, GADD45α triggers demethylation of the promoter proximal DNA by recruiting the nucleotide excision repair (NER) machinery to remove methylated cytosines [143]. 5-Aza-CR is phosphorylated to 5-Aza-CR diphosphate which can be reduced to 5-Aza-CdR diphosphate and subsequently incorporated into DNA. 5-Aza-CdR is phophorylated to 5-Aza-CdR mono- and diphosphate, and then incorporated into DNA. 5-Aza-CdR nucleotide of DNA forms a covalent bond with the DNMT and inactivates these enzymes [144,145]. It has single-agent activity in myeloid malignancies, including myelosplasitc syndrome, acute myelogenous leukemia, and chronic myelogenous leukemia [146,135]. There were also reported that 5-Aza-CdR can selective degrade DNMT1 by a proteasome pathway in certain settings [147]. Zebularine, another derivative of 5-Aza-CR, is converted to 2’-deoxyZebularine 3-phosphate and then is incorporated into DNA. 2’-deoxyZebularine nucleotide of DNA irreversibly inactivates DNMTs by covalently binding to these enzymes [148,149]. Its demethylating and antitumor activity was reported later [148], but its oral bioavailability was low [150]. The drug also been reported preferentially depleted DNMT1, and with some specificity toward cancer cells [149]. Latterly, in order to study 5-Aza-derivatives of cytosine, Byun *et al. *synthesized a intermediate product, 2`-deoxy-N4 [2-(4-nitrophenyl)ethoxycarbonyl]-5-azacytidine (N4-NPEOC-5-CdR), and found that such intermediate product can be activated to 5-Aza-CdR and decrease global and specific DNA methylation like other cytosine analogs in the cells expressing carboxylesterase 1 [151]. The other pyrimidine analogs, 5-fluorocytidine is also a mechanic inhibitor of DNMT, and currently under clinical development [151], but treatment cells with 5-fluorocytidine do not induce the degradation of DNMT1 [147].

One difficulty in using demethylating agents like 5-Aza-CR *in vivo* is the ability to achieve pharmacologically active does without systematic toxicity. The results from *in vivo *studies carried out by Baylin were the first to demonstrate that HDACi sodium phenylbutyrate can synergize with demethylating agent 5-Aza-CR to prevent lung tumor development. In that setting, low-dose demehylating agents combine with HDACi achieve pharmacologically active does without systemic toxicity [110]. The observations that intensification of the 5-Aza-CdR dose markedly increased its antineoplastic acitivity in mouse models of cancer have provide a strong rationale to perform clinical trails using dose intensification of 5-Aza-CdR to maximize the chemotherapeutic potential of this epigenetic agent in patient with cancer [152]. Contrarily, there were also reported that lower doses of 5-Aza-CdR were more effective at inhibiting DNMT *in vitro* and *in vivo* [153,154]. When come to clinical trail, it’s important to optimize the dose-schedule of demethylating agents.

Although the wildly and extensively use of nucleoside DNMTi, but these agents, like current cytotoxic chemotherapy, cause myelosuppression among other side effects that limit exploitation of their demethylating properties, so the development of alternative DNMTi is urgent needed [155].

### 5.3. Dietary DNMTi

During the past years, we have made tremendous progress in understanding of dietary components that prevent or reverse the DNMT induced TSGs inactivation.

**Fig. (4):**
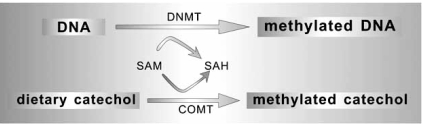
**DNA methylation and its modulation by the COMT-mediated methylated catechols.** Both methylation reactions use the same pools of methyl donor SAM, and both contribute to the formation of SAH as a feedback inhibitor.

It is well known that catechol-o-methyltransferase (COMT) –mediated rapid methylation would not only significantly drain the intracellular pools of SAM, but it would also form equimolal amounts of S-adenosyl-L-homocysteine (SAH), which is the demethylated SAM and is a feedback inhibitor of various SAM-dependent methylation processes (DNMT-mediated methylation). Relying on the knowledge of that various catechol-containing dietary polyphenols are excellent substrates for the COMT-mediated methylation, Lee and colleagues provide a general mechanistic basis for the notion that a variety of dietary catechols (caffeic acid, catechin, epicatechin, (-)-epigallocatechin-3-o-gallate (EGCG), guercetin, fisetin, myricetin and chlorogenic acid) can function as inhibitors of DNA methylation in a complex way. Some catechol-containing dietary polyphenols (such as catechin and epicatechin) have two mechanistic components involved in the inhibition of DNA methylation: One is the directly inhibition of the DNMT (independent of COMT-mediated methylation), and the other is the indirectly inhibition of the DNMT through an increase in SAH formation during the COMT-mediated O-methylation of these dietary chemicals. EGCG, the main polyphenol compound in green tea, whose inhibitive activity is mainly owed to the direct inhibition of the DNMT, is a more potent and efficacious inhibitor of DNMT than other dietary catchols *in vitro*, but under *in vivo* experimental conditions, the former behaves less activity than the latter, because EGCG is an inferior substrate for COMT and has a relatively lower intercellular bioavailability than the latter (Fig. **4**) [156,157]. Reactivation of some methylation-silenced genes (INK4α, RARβ, MGMT, hMLH1) by EGCG was also demonstrated in human cancer cells (colon cancer cell, esophageal cancer cell, prostate cancer cell) [158].

Apple, tea and their products are commonly consumed, which are a rich source of phenolic constituents. Apple products have widely reported to post-translational inhibit the expressing of *DNMT1* and *DNMT3b* [159,160]. Latterly, genistein from soybean has been demonstrated to inhibit DNMT *in vitro*, and Fang M *et al. *associated this with the reactivation of *P16^INK4a^* [161].

Considering that some aberrant DNA methylation is present in early stages of carcinogenesis, there is a possibility that such dietary DNMTi may be useful for cancer prevention and may less effective in therapy. Further studies about the novel DNMTi have been carried out [144].

### 5.4. Others

There are also many less well characterized classes of DNMTi under development, such as hydralazine, RG108, Non-coding RNA (ncRNA), procaine, procainamide and psammaplins. Among non-nucleoside DNMTi currently underway for development, the cardiovascular drug hydralazine has been found with demethylating property through the linkage with immunologic reaction inducing effect and the participation of DNA methylation disorders in immune diseases.

The clinical safety and tolerability have been demonstrated by decades of extensive hydralazine use for hypertensive disorders. For the moment, hydralazine is being evaluated, along with histone deacetylase inhibitors either alone or as adjuncts to chemotherapy and radiation for lung cancer and other refractory solid tumors [155,162]. From the phase II, single-arm study of hydralazine and magnesium valprovate added to the same schedule of chemotherapy on which patients were progressing, Candelaria *et al. *found that demethylating agent hydralazine combined with HDACi magnesium not only reduced global DNA methylation, histone deacetylase activity, and promoter methylation, but also reduced the chemoresistance of the refractory solid tumors [162].

RG108, a novel synthetic small molecule, effectively blocked DNMT *in vitro* and did not cause covalent enzyme trapping in human cell lines. Tumor cells treated with RG108 at a low concentration resulted in a significant DNA demethylation and TSGs reactivation (p16, SFRP1, secreted frizzled related protein-1, and TIMP-3) without detectable toxicity. RG108 also inhibited human tumor cell line (HCT116, NALM-6) proliferation and increased doubling time in culture. Intriguingly, RG108 did not affect the methylation of centromeric satellite sequences. These novel characteristics made RG108 a promising DNMTi for modulation of epigenetic gene regulation [163].

ncRNA is a class of novel useful DNMTi, such as antisense RNA, short interference RNA (siRNA) and microRNA (miR). These DNMT targeting agents are complementary to mRNA of DNMT, induce degradation of the target transcripts and down regulate the DNMT expression [164,165]. Presently, many ncRNA have been demonstrated with demethylating ability, such as MG108, miR-29, DNMT-si (DNMT directing siRNA) [166,167,106].

Local anesthetic procaine and antiarrhythmic drug procainamide, the 4-Aminobezonic acid derivatives, have been shown demethylating activities in cellular assays and in mouse xenograft tumors [168,169], but the procaine must present at a high concentration to be an effective DNMTi in cell-free assays, and it has not been effective in all cell lines tested [170]. The psammaplins inhibit DNMT and HDAC activities in cell-free assays, thus should be evaluated further as both the DNMTi and HDACi [171].

The other less characterized class of nucleoside DNMTi mainly contains procaine, procainamide and psammaplins [168,169,171]. Local anesthetic procaine and antiarrhythmic drug procainamide, the 4-Aminobezonic acid derivatives, have been shown demethylating activities in cellular assays and in mouse xenograft tumors [168,169], but the procaine must present at a high concentration to be an effective DNMTi in cell-free assays, and it has not been effective in all cell lines tested [170]. The psammaplins inhibit DNMT and HDAC activities in cell-free assays, thus should be evaluated further as both the DNMTi and HDACi [171].

### 5.5. DNMTi for Lung Cancer Therapy

The antitumor effect of 5-Aza-CR for lung cancer has been demonstrated by Belinsky *et al*. They found that low dose of 5-Aza-CR could decrease 30% of lung cancer incidence, 50% might be achieved by 5-Aza-CR combined with HDACi sodium phenylbutyrate, which finding might provide a novel clinical strategy to help prevent lung cancer [110]. At the moment, the clinical phase I study of 5-Aza-CdR in combination with vaproic acid (VA) in patients with NSCLC is undergoing. And the results show great promise in NSCLC therapy [172].

As 5-Aza-CR, most DNMTi were first studied in myelodysplastic syndrome, and then were used for the solid tumor trials, therefore the development of DNMTi for lung cancershould take much longer than expected. The studies of DNMTi for lung cancer therapy are summerised in Table **2**.

The L-selenomethionine, a nutrient demonstrated to reduce by half the incidence of expected lung cancer, may act partially through inhibition of DNMT [91,173]. And nonsteroidal anti-inflammatory drugs (NSAIDs) have been shown to exhibit potent anticancer effects *in vitro* and *in vivo*. Mithramycin A (MMA) is known to be a GC and CG-rich DNA binding agent. Rou *et al. *found that this kind agent could serve as a DNMT1 inhibitor. When the highly metastatic CL1-5 lung cancer cells treated with MMA, the metastatic and invasion ability of the cell would be reduced, these results indicated a new agent for advanced lung cancer therapy [174]. Latterly, Pan *et al. *first demonstrated that NS398, a NSAID, would inhibit lung cancer cell invasion, and the mechanism was NS398 demethylating the secreted protein acidic and rich in cysteine (*SPARC*) gene in lung cancer [175].

Antisense oligonucleotide was under clinical trails for lung cancer [176]. MG98 was an antisense oligonucleotide, which hybridized to the 3’-untranslated region (UTR) of DNMT1 mRNA and caused degradation of the transcript [15,177]. As an allosteric inhibitor, MG98 inhibit DNMT may also through the competition with the substrates for DNMT [166]. MG98 and siRNA directing to DNMT1 mRNA induced lower DNMT1 level and reexpression of *RASSF1A, CDKN2A *in culture lung cancer CALU-6, and A549 cells have been demonstrated [101]. MiR-29 represents a class of naturally occurring small noncoding RNA molecules, which could bind to the 3’-UTR of target mRNAs and cause a block of translation [164]. The evidences that enhanced expression of MiR-29s in lung cancer cell lines A549 and H1299 restored normal silenced TSG, such as *FHIT* and *WWOX*, and inhibit tumorigenicity both *in vitro* and *in vivo*, resulted in a conclusion that expression of the MiR-29s (29a, 29b, 29c) was inversely correlated to DNMT3A and -B expression in lung cancer tissues and that MiR-29s directly target the 3’-UTRs of *Dnmt3a* and* -3b* [167]. Oridate *et al. *used siRNA to disrupt the expression of DNTM1 in human NSCLC A549 cells and found the decreased DNMT1 level was accompanied by suppression of cell proliferation and clone-forming ability. The mechanism of this may be due to the up-regulated cyclin dependent kinase inhibitor P21. Their study suggested that the siRNA approach could be used to disrupt effectively DNMT1 activity and lung cancer cell growth [168].

Although the latest studies are concerned about the effect of demethylation on lung cancer, but one must be born in mind that down regulation of DNMT1 leads to a decrease in genomic instability [97]. In a mouse model for sarcomas, *Dnmt1*-deficient mice developed sarcomas at an earlier age [178]. Another study showed that mice carrying a hypomorphic and a null allele of *Dnmt1* developed aggressive T cell lymphomas [179]. However, some DNMT silenced genes may do well in the lung cancer therapy. Staub *et al*. reported that although DNMT induced HSulf-1 silence in ovarian cancer, but just this silenced Hsulf-1 sensitized the cancer to conventional first-line therapies. In this case, the DNMT played a positive role in therapy [180]. The potentially adverse effects of DNA hypomethylation have led investigators to suggest that the most effective clinical use of DNMTi may be to combine these drugs, in a temporary and acute fashion, with secondary agents whose efficacy is enhanced by DNA hypomethylation [181]. Taken all data together, there were dual faces of DNMT, tumor induction and suppression, therefore when the DNMT is selected as therapeutic target. A fine equilibrium must be well done before a clinic trial is launched.

## 6. CONCLUSION AND PERSPECTIVES

Epigenetic alterations are, at least, if not more important than genetic defects for the development and progression of lung cancer. In earlier days, it was thought, mistakenly, that alterations of DNA methylation occurred only as a result of cancerization. Because alternations of DNA methylation occur even in the precancerous stage before establishment of cancer and determine the clinicopathological characteristics of the developing malignancies. It is obvious that they are not a secondary result of cancerization. The role of DNMT-mediated epigenetic alterations in lung cancer development has been the focus of increasing interest in recent years. These epigenetic abnormalities are present in almost all cancers and, alongside genetic changes, drive lung cancer progression [26].

A series of events occur during lung tumorigenesis and the corresponding medical actions taken are summarized in Fig. (**5**). Several breakthroughs have been achieved [2], especially in the DNA methylation and histone code realm. The proposals of epigenetic biomarker, epigenetic silencing, methylation profiling, histone coding, self-reinforcing epigenetic cycle model, epigenomics, epigenetic profile, clearly reflect the intensive investigation on epigenetic regulation in lung tumorigenesis. Nevertheless, a number of key questions remain unanswered, such as the definite role of DNMTs in lung tumorigenesis; the role of other epigenetic regulations; the mechanism of DNA methylation ‘converses’ with other histone modification and reinforces suppression function mutually and the nature of DNMT targeting specific gene. Further studies about DNMT induced epigenetic regulation is needed, and will offer more perspectives in prevention, detection, diagnosis and post-treatment assessment of lung cancer and other malignancies.

## Figures and Tables

**Fig. (5). Profile of lung tumorigenesis and medical prevention and therapies.**
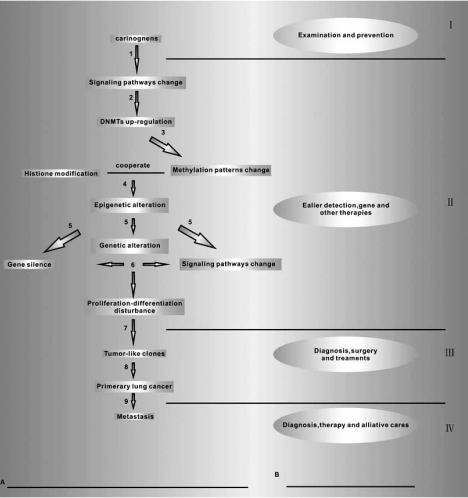
**A.** Profile of lung tumorigenesis I: Carcinogens (such as tobacco components) stimulate certain signaling pathways in lung cells; II: DNMT expression induced by certain signaling pathway (e.g. Ras-Ap1); III: Up-regulated DNMT changes the normal methylation patterns; IV: With the help of other factors, the DNA methylation teams up with histone modification to cause the epigenetic alterations; V: Epigenetic regulation induces genetic changes (e.g. 3p deletion), gene silence (TSGs), certain cancer related signaling pathway alterations (Wnt); VI:Genetic alterations, signaling pathway change and gene silence is correlated & cooperated to disturb the proliferation-differentiation balance (assisting proliferation & blocking differentiation); VII: The non-arrest cell-cycle leads to the formation of tumor-like clones; VIII: The tumor-like clones turn into primary lung cancer; IX: The primary lung cancers begin to metastasis and transfer to other tissues. **B.** Steps are taken to prevent and/or treat lung cancer during lung tumorigenesis. I: Medical examination and prevention; II: The earlier detection, gene and others therapies; III: Diagnosis, surgery and treatments; IV: Diagnosis, therapies and palliative cares.

**Table 1:** TSGs that are Commonly Methylated in Lung Cancer

TSGs	Function	References
*BRACA1*	DNA damage repair	[74]
*BVES *	Cell shape and movement	[75]
*Caspase-8*	Apoptosis	[76]
*CDH1(E-cadhein)*	Cell-cell adhesion	[77,75,78]
*CDH13(H-cadhein)*	Cell adhesion	[79,80]
*CDKN2A(p14)*	cyclin-dependent kinase inhibitor	[63]
*DAPK1*	Interferon-induced apoptosis	[75]
*Estrogen receptor*	Growth control	[81]
*FHIT*	Cell apoptosis	[82]
*hsRBC*	DNA repair	[83]
*ING1 *	Cell growth and apoptosis	[84]
*KCNH5*	Membrane voltage gate	[75]
*KISS1*	Chemotaxis and invasion	[85]
*MGMT*	DNA repair	[75]
*MTHFR*	DNA synthesis and repair	[63]
*MYO18B*	Growth control	[86]
*P16*	Cell cycle regulation	[79]
*P15/INK4b*	Cell-cycle control	[87]
*P53*	Cell cycle and differentiation	[88]
*PTEN*	Cell movement and adhesion	[89]
RARB	Signal transduction	[75]
*RASSF1A*	Cell cycle arrest	[63,75]
*RB*	Cell-cycle control	[90]
*RUNX*	Transcription factor	[91]
*SOCS-3*	Growth control	[92]
*SOCS-7*	Growth control	[93]
*STK11 *	Signal transduction	[94]
*WWOX*	Transcription regulation, protein degradation	[82]
*TMS1*	Apoptosis	[95]
*T*(brachyury homologue)	Transcription regulation	[96]

**Table 2:** The Studies of DNMTi for Lung Cancer Therapy

Drug	Phase	Cancer	Results	References
5-Aza-CR	Approved by FDA	Mainly for MDS	improve overall response rates, time to leukemic progression, and quality of life	[182]
5-Aza-CdR	Approved by FDA	Mainly for MDS	More effective than 5-Aza-CR	[ http://www.fda.gov/CDER/Offices/OODP/whatsnew/decitabine.htm ]
5-Aza-CR	I/II	Recurrent NSCLC	On-going	[ http://clinicaltrials.gov/ct2/show/NCT00387465 ]
5-Aza-CdR	I	Metastatic NSCLC	5-aza-CdR in combination with valproic aci is well tolerated and shows promise in gene demethylation	[172]
ATRA	III	AML, MDS, NSCLC, breast cancer, glioblastoma and melanoma	show promising efficacy in combination with the Valproic acid	[183]
Fazarabine	II	Advanced NSCLC	has no demonstrable activity in metastic NSCLC patients	[184]
Zebularine	Preclinical	AML	inhibits cell proliferation, arrests cells at G2/M, and induces apoptosis	[185]
EGCG	Preclinical	Lung cancer	Induce apoptosis of lung cancer cell lines A549 and ChagoK-1	[186]
L-selenomethionine	Preclinical	Lung cancer	Reduce incidence of lung cancer	[91]
Hydralazine	II	Lung cancer and other solid tumor	Decrease the methylation and reduce the chemoresistance of lung cancer and other refractory solid tumor	[162]
MG98	I	Lung cancer and other advanced solid malignancies	Suppression of DNMT1 expression	[187]
MiRNA-29	Preclinical	Lung cancer	Restored silenced TSGs in A549 and H1299 lung cell lines	[167]
DNMT1-siRNA	Preclinical	Lung cancer	suppression of cell proliferation and clone-forming ability	[106]
MMA	Preclinical	Lung cancer	Inhibit invasion and metastasis	[174]

## ABBREVIATIONS

5-Aza-CR: = 5-Aza-cytidine
5-Aza-CdR: = 5-Aza-2’-deoxycytidine
AC: = Lung adenocarcinoma
AML: = Acute myeloid leukemia
ATRA: = All-trans-retinoic acid
ATRX: = Cysteine alpha thalassemia retardation on the X rich zinc finger DNA binding motif
BVES: = Blood vessel epicardial substance
CAV1: = Caveolin-1
CDH1: = E-cadherin
CDH13: = H-cadherin
CDKN2A: = P16/INK4a
COMT: = Catechol-o-methyltransferase
DAP: = Death-associated protein
DAPK: = Death-associated protein kinase
DNMT: = DNA methyltransferase
DNMTi: = DNA methyltransferase inhibitor
EGCG: = (-)-epigallocatechin-3-o-gallate
FDA: = Food and drug administration
FHIT: = Fragile histidine triad
GADD45α: = Growth arrest and DNA damage inducible, alpha
GK: = Gly-lys sequence
H3K4: = H3 lysine 4
HDAC: = Histone deacetylase
HDACi: = Histone deacetylase inhibitor
HKMT: = Histone-lysine methyltransferase
hsRBC: = Human SRBC gene
ING1: = Inhibitor of Growth 1
LSH: = Lymphoid-specific helicase
MBD: = Methyl-CpG binding protein
MDS: = Myelodysplastic syndrome
mCpG: = Methyl-CpG site
MeCP: = Methyl CpG binding protein
MGMT: = O-6-methylguanine-DNA methyltransferase
MiR: = MicroRNAs
MM: = Malignant mesothelioma
MMA: = Mithramycin A
NcRNA: = Non-coding RNA
NER: = Nucleotide excision repair
NLS: = Nuclear localization signal sequence
NSCLC: = Non-small cell lung cancer
PCNA: = Proliferation cell nuclear antigen
PDB: = Proliferating cell nuclear antigen binding domain
PHD: = Polybromo homology domain
PWWP: = Pro-Trp-Trp-Pro tetrapeptide sequence
RARB: = Retinal acid receptor beta
RASSF: = Ras association domain family
rDNA: = Ribosomal DNA
rRNA: = Ribosomal RNA
SAH: = S-adenosyl-L-homocysteine
SAM: = S-anenosyl-L-methionine
SC: = Squamous carcinoma
SCLC: = Small cell lung cancer
siRNA: = Short interference RNA
SPARC: = Secreted protein acidic and rich in cysteine
STK11: = Serine/threonine kinase
TF: = Transcription factors
TRD: = Aspartic acid transfer RNA
TSG: = Tumor suppressor gene
VA: = Vaproic acid
UTR: = Untranslated region
WWOX: = WW domain containing oxidoreductase
